# Report of the Pennsylvania Hospital for the Insane, for the Year 1851

**Published:** 1852-03

**Authors:** 


*Report of the Pennsylvania Hospital for the Insane, for the year* 1851. By Thomas S. Kirkbride, M. D., Physician to the Institution.

Dr. Kirkbride's *eleventh* annual report presents a highly interesting picture of the noble institution under his charge. The following points in connection with the history of the hospital during the last year, deserves notice.

"For several months, during the past year, the whole house was inconveniently crowded, but the general good health which then prevailed enabled us to receive all the cases that were brought to the Hospital, although much difficulty was often experienced in accommodating them. As a large proportion of the people of Pennsylvania have always been accustomed to look to the Pennsylvania Hospital for relief for their friends when afflicted with insanity, it was felt to be a duty, as far as possible, to receive all whose condition rendered their removal from home important, or who were likely to suffer from the want of prompt treatment. The prospect for the State provision for the insane being soon available, was also another reason for our being willing, for a short period, to be somewhat incommoded by too large a number of patients, and we now have reason to believe that our present apartments will hereafter prove ample to accommodate all applicants for admission.

Of the patients discharged during the year 1851, were

| | |
|---|---|
| Cured | 107 |
| Much improved | 13 |
| Improved | 32 |
| Stationary | 23 |
| Died | 26 |
| Total | 201 |

Of the patients discharged "cured," fifty-two were residents of the Hospital not exceeding three months; twenty-six between three and six months; twenty-three between six months and one year; and six for more than one year.

Of those discharged "much improved," three were under treatment less than three months; five between three and six months; two between six months and one year; and three for more than one year.

Of the "improved," nine were under care less than three months; five between three and six months; eleven between six months and one year; and eight for more than one year.

Of those discharged and reported "stationary," six were under care less than three months; four between three and six months; four between six months and one year; and nine for a longer period than one year.

Sixteen males and ten females have died during the year. Of these deaths, eight resulted from acute mania; one from acute dementia; five were cases of organic disease (softening) of the brain, of which the mental affection was only a symptom; one was from epilepsy; two from pulmonary consumption; three from dysentery; one from chronic ulceration of the throat; one from the exhaustion produced by a long continued refusal of food; one from suicide; one from cancer; and two from old age.

Of these deaths, eight occurred within less than a fortnight after their admission; while two were of patients who had been thirty-six years residents of the Hospital, and one had been more than twenty-five years.

Several of the cases of acute mania were undoubtedly injured by the journey to the Hospital during the existence of the acute symptoms, which often cannot without difficulty be distinguished from those of inflammation of the brain. While this doubt exists, the patient had better be retained at home, and the probabilities of ultimate recovery are not lessened by such a course.

Of the patients who died, ten were admitted for mania; six for melancholia; and ten for dementia."

"The most important improvements made during the year, have been the erection of a new Museum and Reading-Room, increased means for a supply of water, with a change in the mode of raising it to the dome of the building, and additional apparatus for security from accidents by fire.

In addition to these improvements, a handsome summer-house has been erected on the mound in the ladies' pleasure-grounds. This mound, it may be recollected, is in a portion of the grounds from which, formerly, the view was very limited. The mound is about 80 feet in diameter at its base, 11 feet high, terraced and planted with shrubs and flowers, and, from the new summer-house on its summit, the view is one of the most extensive and pleasant to be found on the premises, without being at all deficient in the requisite degree of privacy.

The pleasure railroad, formerly in the lawn adjoining the main front of the centre building, has been removed to the woods, in the grounds appropriated to the female patients. This position for it is more desirable in every respect; with a much greater degree of privacy for all, it has now become accessible to a large class of patients who before had rarely, if ever, used it; while the fine trees, among which it is placed, make the exercise quite admissible at all hours, even in the warmest weather. The only objection to this arrangement is that, as now situated, the road can be used only by the female patients. With our present increased numbers, however, it will probably soon be deemed expedient to erect one of a similar character, on some part of the men's grounds, as it is a valuable means of exercise and amusement, highly enjoyed by a large number of patients.

The main culvert, into which all the branch culverts enter, and which formerly terminated a few feet beyond the western wall of the pleasure-grounds, has been extended across the adjacent meadow, a distance of 229 feet, so as to empty directly into Mill Creek, instead of doing so by circuitous route, as had been previously the case.

A bath-room over a vestibule on the north side of the physician's residence, has been completed; and that building supplied with water by a connection with the reservoirs in the dome of the Hospital."

"*The Association of Medical Superintendents of American Institutions for the Insane.*—Among the pleasant incidents of the past year, must be noted the very gratifying visit, which this Hospital in common with other institutions for the insane in this vicinity, received from the members of this Association during its recent meeting in Philadelphia. Founded in 1844, when its first meeting was held in Philadelphia, it now numbers among its associates nearly all the members of the medical profession in America who are devoting themselves to the care and treatment of the insane. It has held annual meetings in various parts of the United States, and its members have visited and examined a number of the institutions for the treatment of mental diseases.

Such a body of men, possessing so large an amount of practical knowledge on this interesting subject, so capable of judiciously criticising what is defective and appreciating what is commendable in every department of such establishments, cannot but always be most welcome guests to all who are in any way concerned in their management, and anxious to profit by the experience of others. It can scarcely admit of a doubt but that it is the interest of every institution for the insane, on the continent, to be regularly represented at the meetings of the Association."

